# Meta-Analysis of Pathway Enrichment: Combining Independent and Dependent Omics Data Sets

**DOI:** 10.1371/journal.pone.0089297

**Published:** 2014-02-28

**Authors:** Alexander Kaever, Manuel Landesfeind, Kirstin Feussner, Burkhard Morgenstern, Ivo Feussner, Peter Meinicke

**Affiliations:** 1 Department of Bioinformatics, Institute of Microbiology and Genetics, Georg-August-University, Göttingen, Germany; 2 Department of Plant Biochemistry, Albrecht-von-Haller-Institute for Plant Sciences, Georg-August-University, Göttingen, Germany; University of Edinburgh, United Kingdom

## Abstract

A major challenge in current systems biology is the combination and integrative analysis of large data sets obtained from different high-throughput omics platforms, such as mass spectrometry based Metabolomics and Proteomics or DNA microarray or RNA-seq-based Transcriptomics. Especially in the case of non-targeted Metabolomics experiments, where it is often impossible to unambiguously map ion features from mass spectrometry analysis to metabolites, the integration of more reliable omics technologies is highly desirable. A popular method for the knowledge-based interpretation of single data sets is the (Gene) Set Enrichment Analysis. In order to combine the results from different analyses, we introduce a methodical framework for the meta-analysis of p-values obtained from Pathway Enrichment Analysis (Set Enrichment Analysis based on pathways) of multiple dependent or independent data sets from different omics platforms. For dependent data sets, e.g. obtained from the same biological samples, the framework utilizes a covariance estimation procedure based on the nonsignificant pathways in single data set enrichment analysis. The framework is evaluated and applied in the joint analysis of Metabolomics mass spectrometry and Transcriptomics DNA microarray data in the context of plant wounding. In extensive studies of simulated data set dependence, the introduced correlation could be fully reconstructed by means of the covariance estimation based on pathway enrichment. By restricting the range of p-values of pathways considered in the estimation, the overestimation of correlation, which is introduced by the significant pathways, could be reduced. When applying the proposed methods to the real data sets, the meta-analysis was shown not only to be a powerful tool to investigate the correlation between different data sets and summarize the results of multiple analyses but also to distinguish experiment-specific key pathways.

## Introduction

High-throughput omics platforms, such as mass spectrometry (MS) based Metabolomics and Proteomics or DNA microarray or RNA-seq-based Transcriptomics, allow the comprehensive analysis of an organism's reaction under different experimental conditions [1]–[5]. A current major challenge in systems biology is the combination and integrative analysis of the large data sets obtained from these platforms [6]–[8]. A single data set usually contains the intensity/expression profiles (intensities for all measured samples) of thousands of features, such as different ion species in MS or spots in DNA microarray analysis. After individual preprocessing of each data set, which includes the statistical analysis, ranking, or filtering of features according to the relevance of their profiles [9]–[11], the features have to be assigned to known biological entities [12], such as metabolites, genes, or proteins. Especially in MS-based Metabolomics, a major bottleneck is the identification of metabolites in non-targeted experiments [13]. In many applications, the putative monoisotopic masses of measured ion species cannot unambiguously mapped to metabolite entries in public databases. The integration of data from other omics platforms which provide a more reliable mapping, such as DNA microarrays, can significantly support the metabolite identification in this case. After annotation, the results are usually interpreted in the context of current knowledge, e.g. known biochemical pathways or processes [14]–[16]. A popular method for this knowledge-based interpretation of single data sets is the Gene Set Enrichment Analysis [17] or Overrepresentation Analysis [18], [19]. Many similar approaches have been developend and the methodology was transferred to other omics platforms [20]–[23]. In general, the enrichment analysis is based on sets of entities, e.g. pathways with associated metabolites, and results in a list of relevant sets which are enriched in high-ranking features (in comparison to all features in the data set). In most methods, the enrichment level of a single set is expressed as p-value. Modelling metabolic pathways as well-defined sets of biological entities, e.g. metabolites, enzymes, and corresponding genes, has shown to be a powerful approach to interpreting complex omics data sets. Furthermore, the concept of pathways associated with different types of biological entities facilitates the joint analysis of different data sets [24].

The combination of results from different studies sharing the same experimental design in terms of null and alternative hypothesis (meta-analysis) is a central task in various statistical applications [25]–[27]. In case of the combination of independent p-values, Fisher's method [28] or Stouffer's method [29], also known as normal, Z-method, or Z-transform test, are often applied. For dependent p-values and known covariances, in [30] an extended version of Fisher's method was proposed (Brown's method). In order to increase statistical power, meta-analysis has been applied to Pathway Enrichment Analysis (Set Enrichment Analysis utilizing pathways as sets) in the context of cancer studies [31]. The proposed methods were focused on the combination of independent p-values based on DNA microarray data. In contrast, we introduce a general methodical framework for the meta-analysis of multiple dependent or independent data sets resulting from different omics platforms applied to Pathway Enrichment Analysis. In order to cope with dependent data sets, such as obtained from the same biological samples analyzed by MS in negative and positive ionization mode, the framework utilizes a covariance estimation procedure based on the nonsignificant pathways in single data set enrichment analysis. The framework is applied and evaluated on two Metabolomics MS data sets [32] and two Transcriptomics DNA microarray studies [11] in the context of wounding of *Arabidopsis thaliana*. The main focus of this exemplary meta-analysis lies on the enhancement of MS based Metabolomics results by means of the microarray studies.

## Materials and Methods

### Data sets and preprocessing

For application and evaluation of the meta-analysis, two Metabolomics MS data sets (M1 and M2) [11] and two Transcriptomics DNA microarray data sets (T1 and T2) [32] were used (see Table 1 and Dataset S1 for details). All studies investigate the wounding of *Arabidopsis thaliana* wild type and the jasmonate-deficient *dde* 2-2 mutant plants [33], the experimental designs comprise conditions for control plants as well as plants harvested at different times after wounding (see Table 1). The two Metabolomics data sets derive from an Ultra Performance Liquid Chromatography (UPLC) analysis coupled to a Time-Of-Flight (TOF) MS detection. With this method, the non-polar extraction phase of one set of samples was analyzed in positive and negative ionization mode. Since some metabolites may have been measured in both ionization modes following different (partially unknown) ionization rules [34], the level of dependence between both data sets is not clear. In case of the MS data sets, a single feature corresponds to a particular ion species, which is characterized by an exact mass-to-charge ratio and a retention time. A single metabolite may be represented by multiple features, e.g. corresponding to different adduct formations and isotopologues. The features in the microarray data sets correspond to different spots on the array containing DNA probes that match a particular sequence. Also in this case, a single transcript may be represented by multiple features corresponding to particular sequences of the respective gene. The feature profiles of all data sets were ranked separately utilizing a signal-to-noise ratio (similar to the method described in [9], see TechnicalDescription S1).

**Table 1:** Overview on data sets.

Label	Number of features	Times	Platform	Ionization mode	Reference
M1	24796	0.5 h, 2 h, 5 h	Mass spectrometry	negative	[11]
M2	23325	0.5 h, 2 h, 5 h	Mass spectrometry	positive	[11]
T1	25392	1 h	DNA microarray	-	[32], E-ATMX-9
T2	25392	3 h	DNA microarray	-	E-MEXP-1475

The table gives an overview on the four data sets used for evaluation and application. The third column (Times) summarizes the different points in time when the wounded plants were harvested in the respective experiment. The T1 and T2 data sets can be obtained from the ArrayExpress [44] website.

### Pathway enrichment analysis

The ranked features were mapped to the pathway entries in AraCyc [35] and the Arabidopsis-specific pathways in the Kyoto Encyclopedia of Genes and Genomes (KEGG) database [14] (see TechnicalDescription S1). In case of the Metabolomics MS data sets, all potential monoisotopic masses were calculated per feature based on the ionization rules and number of isotopes used in [11] and mapped to the metabolite masses in the databases. In case of the Transcriptomics DNA microarray data, the features were mapped to the *A. thaliana* genes utilizing their CATMA IDs [36]. Based on the mappings, a set of feature ranks was extracted for each pathway and data set. In order to test for an over-representation of high-ranked features, a p-value was calculated for each set of ranks (pathway) utilizing a one-sided Kolmogorov-Smirnov (KS) or Wilcoxon rank-sum test (also known as Mann-Whitney U test) [21]. In case of the KS test, the empirical distribution of ranks in a given set is compared to the distribution of ranks in the respective data set. In case of the rank-sum test, the sum of feature ranks within a given set is evaluated. Especially for Gene Set Enrichment Analysis of DNA microarrays, many methods have been published [20]. Most of these methods are based on KS-like or average gene-specific statistics. For a general meta-analysis and in order to combine the Metabolomics and Transcriptomics data sets in a robust way, we decided to utilize the rank-based KS and rank-sum test. However, more specialized methods for the pathway-specific p-value calculation may be employed as well. The resulting p-values for the dependent Metabolomics data sets were used for the covariance estimation (see corresponding section). The covariances between both Transcriptomics data sets and between the Metabolomics and Transcriptomics data sets, which were obtained from independent biological samples, were set to zero.

### Meta-analysis of p-values

In statistical meta-analysis, the most common methods for combining independent p-values from related tests are Fisher's [28] and Stouffer's method [29]. In Fisher's method, the meta-p-value is calculated based on a chi-squared distribution (see TechnicalDescription S1). In Stouffer's method, the test statistic is the sum of p-values transformed into normally distributed random variables (standard normal deviates). For dependent p-values, a powerful approach is Brown's method [30], which is an extension of Fisher's method based on a scaled chi-squared distribution and modified degrees of freedom utilizing a known covariance matrix for standard normal deviates. The given p-values can be transformed into standard normal deviates by means of the inverse cumulative distribution function of the standard normal distribution. The covariance matrix of the standard normal deviates can also be utilized in order to extend Stouffer's method to dependent p-values.

### Estimation of covariances

In most applications with dependent data sets, the covariance matrix is not known and has to be estimated. In our proposed procedure, the pairwise covariance between two data sets is estimated based on the standard normal deviates of the pathway-specific p-values, which were obtained for each single data set in Pathway Enrichment Analysis. This estimation is expected to be biased by the alternative hypothesis since the similar or same experimental setup of the data sets imposes a certain dependence and significant pathways associated with very low p-values will strongly influence the results. In order to minimize this bias in the estimation of the pairwise covariance between two data sets, a parameter 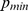 is introduced and only pathways with p-values in the range 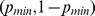 are considered. This procedure leaves out significant pathways for which the null hypothesis is likely to be rejected for at least one of the data sets. Instead of directly estimating the sample covariance of the transformed p-values in this range (which would again be biased because of the range restriction), Pearson's correlation coefficient is used.

## Results

The Pathway Enrichment Analysis, the transformation of pathway-specific p-values into standard normal deviates, the estimation of covariances for dependent data sets, and the meta-analysis based on the previous results were applied and evaluated on the four Metabolomics/Transcriptomics data sets (see previous section). First, in order to check the distribution of transformed p-values, the histograms of the standard normal deviates were inspected. Because of significant pathways which are highly relevant in this context, the p-values are expected to be not fully uniformly distributed, which may result in a distribution of transformed p-values that deviates from the standard normal distribution. In this case, the p-values/normal deviates should be corrected for significance analysis. Second, the performance of the introduced method in reconstructing simulated data set correlations was evaluated for different 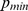 values. This performance was not clear, since the proposed correlation estimation includes several complex steps, such as the mapping of a proportion of feature ranks to pathways of different size, the calculation and restriction of p-values, and the transformation into normal deviates. Additionally, the 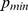 parameter might have a strong influence on the results. Therefore, another objective of the simulation studies was the identification of an appropriate parameter value for the real data sets. Third, the correlation estimation and meta-analysis were applied to all four real data sets. All data sets, containing the annotation information from the pathway mapping, and the results from Pathway Enrichment Analysis are available as comma-separated-values files (see Dataset S1 and Table S1). The source code of functions for the meta-analysis of p-values can be found in File S1.

### Distribution of standard normal deviates

**Figure 1:**
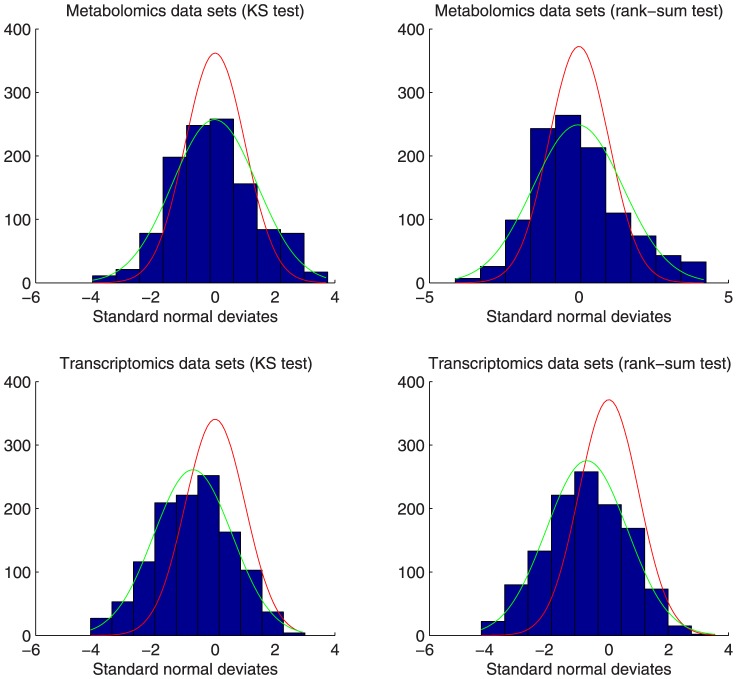
Histograms of standard normal deviates for the Metabolomics and Transcriptomics data sets. For the p-value calculation, the Kolmogorov-Smirnov (KS) and rank-sum tests were utilized. The p-values were restricted to the range 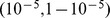. The red graph represents the expected density assuming the standard normal distribution. The green graph shows the expected density assuming a normal distribution with the sample mean and standard deviation as parameters. The histograms for both tests are similar and confirm the normal-like distribution of deviates. In both cases however, the sample standard deviation is higher than the unit standard deviation used for the transformation. Additionally, the sample mean for the combined Transcriptomics data sets is smaller than zero.

Figure 1 shows the histograms of the transformed p-values (standard normal deviates) from Pathway Enrichment Analysis for the two Metabolomics and two Transcriptomics data sets within the p-value range 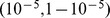. The histograms for the KS and the rank-sum test are similar and confirm the normal-like distribution of deviates. In both cases however, the sample standard deviation is higher than the unit standard deviation used for the transformation. Additionally, the sample mean for the combined Transcriptomics data sets is smaller than zero. This difference may be caused by pathways which are directly or indirectly influenced by the experimental setup. Although the highly significant pathways with p-values below the threshold 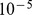 were left out, many other pathways are expected to be indirectly affected by the wounding process. Another explanation would be the dependence of feature ranks used for p-value calculation, e.g. introduced by the dependence of different microarray spots representing the same gene or by gene-gene correlations [17]. In order to eliminate the observed bias, the p-values were restandardized [37] for significance analysis by means of the sample mean and sample standard deviation of observed normal deviates per data set and retransforming of the standardized deviates into corrected p-values. This is a conservative correction because the observed bias also includes the pathways which are directly influenced by the wounding process.

### Estimation of data set correlation

In simulated studies (see TechnicalDescription S1 for details), the correlation estimation was evaluated by calculating the pairwise Pearson correlation coefficients between all four data sets and a copy of the respective data set with different percentages of feature ranks randomly permuted. For each original and permuted data set, the p-values were calculated for all pathways using the KS or rank-sum test. The correlation coefficient between each original and permuted data set was computed based on the respective standard normal deviates (not restandardized) and the restriction of p-values utilizing different parameter values 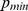. As measurement of the introduced artificial correlation, the correlation coefficient between the feature ranks of each data set and the permuted ranks (feature rank correlation) was calculated and averaged, respectively. The whole procedure was repeated for negative correlation by randomly permuting a percentage of the inverted original feature ranks per data set.

**Figure 2:**
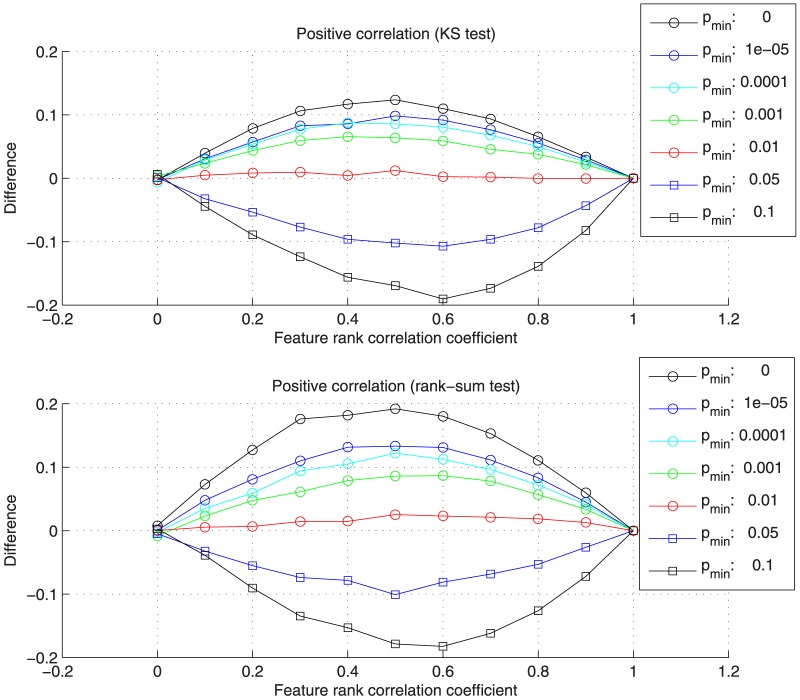
Differences between the reconstructed correlation coefficients from pathway enrichment and the introduced positive feature correlation. The differences were calculated for different 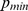 values and the Kolmogorov-Smirnov (KS) and rank-sum test. The best reconstruction, corresponding to differences near zero, can be observed for 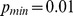.

**Figure 3:**
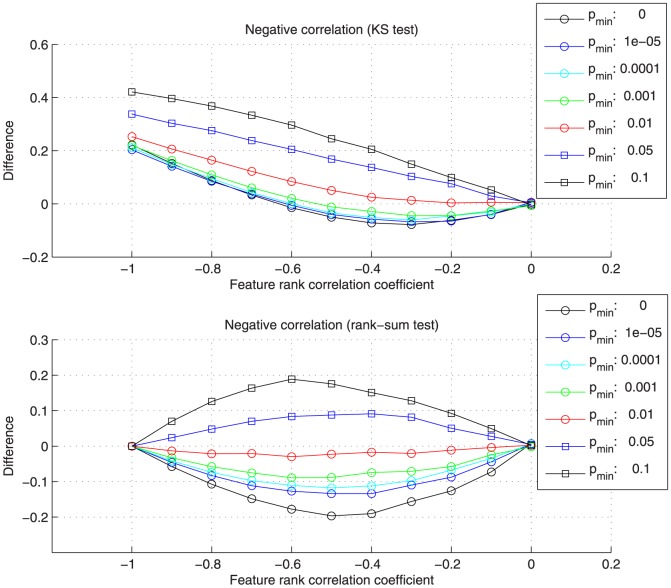
Differences between the reconstructed correlation coefficients from pathway enrichment and the introduced negative feature correlation. The differences were calculated for different 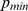 values and the Kolmogorov-Smirnov (KS) and rank-sum test. The best reconstruction, corresponding to differences near zero, can be observed for 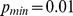. The KS test is not able to fully reconstruct strong negative feature correlations.

Table S2 shows the average results over all data sets in detail. Figure 2 and 3 summarize the differences between the reconstructed correlation coefficients from pathway enrichment and the introduced positive or negative feature rank correlation. In comparison to the average feature rank correlation coefficients (x-axis), the absolute correlation is overestimated for low 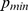 values and underestimated for high values. A 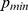 value of 0.01 results in the best reconstruction of data set correlation, the absolute difference between the correlation coefficients from pathway enrichment and the feature rank correlation is close to zero for both tests. In case of the observed overestimation for low 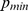 values, the relevant pathways, which are associated with many top-ranking features, are assigned a low p-value, even when randomly permuting some of the features, and have a high influence on the correlation estimation. In case of the underestimation for high 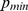 values, the introduced correlation over all features and pathways cannot be fully recovered when restricting the range of p-values and number of utilized pathways too much.

For the KS test and small negative feature rank correlations, the estimated coefficients from enrichment are considerably larger, e.g. showing a difference between 0.2 and 0.4 in case of a feature rank correlation of −1 (see Figure 3). This can be explained by the non-symmetric properties of the one-sided KS test. A set enriched in both high-ranking and low-ranking features would receive a low p-value when performing the one-sided KS test on the original as well as the inverted ranks. The rank-sum test, on the contrary, would result in an average p-value in both cases because the sum of ranks in the set is near the expected value. For a 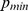 value of 0.01 and negative correlation, the KS test is still able to reconstruct feature rank correlation coefficients between 0 and −0.3 with a difference near zero.

For the correlation estimation between the two dependent Metabolomics data sets, a 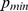 value of 0.01, which showed the best reconstruction in the simulations, was utilized. The estimation resulted in relatively small coefficients, 0.12 (KS test) and 0.08 (rank-sum test).

### Meta-analysis of pathway enrichment

**Figure 4:**
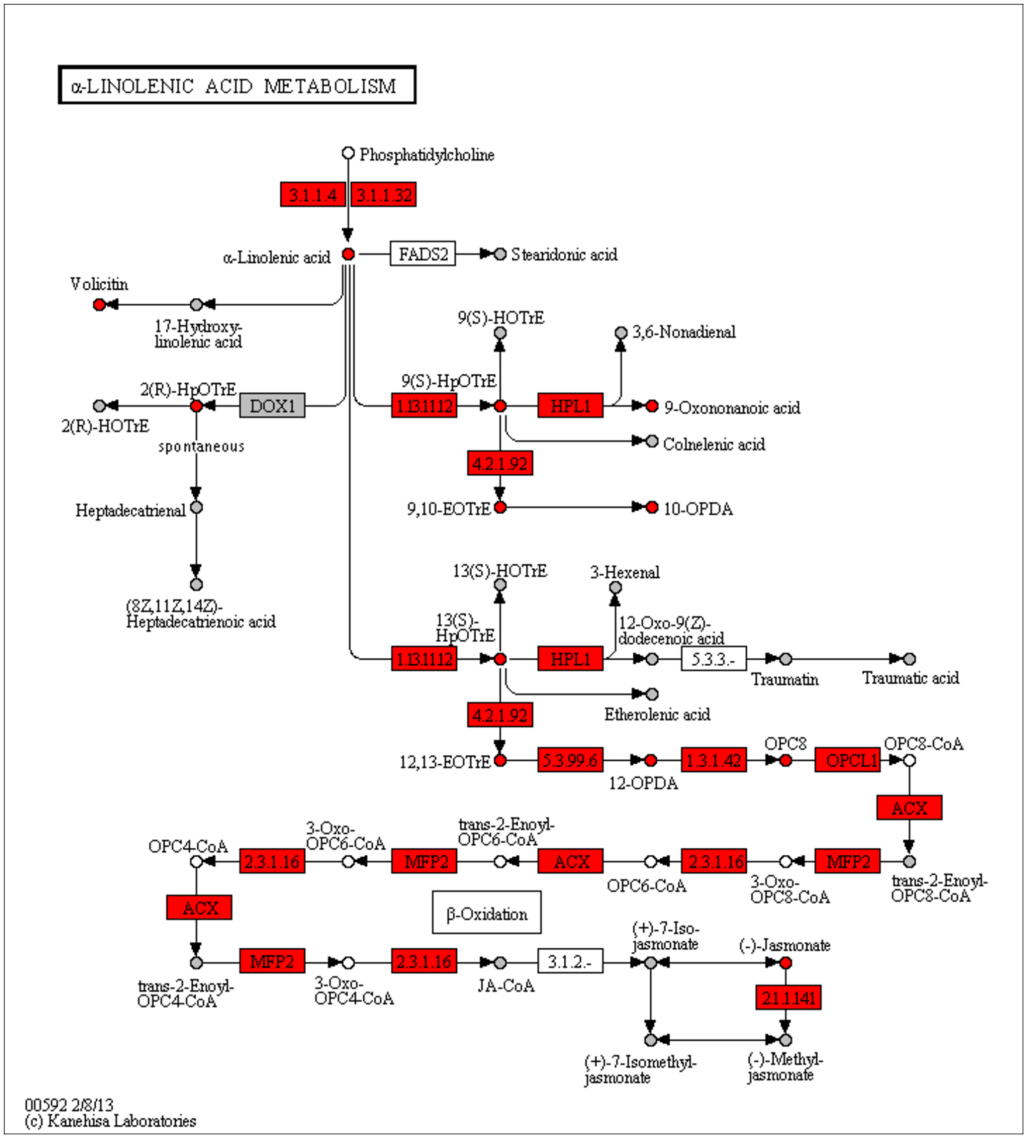
Pathway map of the alpha-linolenic acid metabolism (KEGG) with marked entries. Entries mapped to features from all data sets are marked in gray, selected entries from Tables 5, 7, 9, and 11 are marked in red.

Tables 2 and 3 show the results from meta-analysis of pathway enrichment utilizing Brown's and Stouffer's extended method integrating the correlation estimation for the Metabolomics data sets. The pathways are sorted according to the False Discovery Rate (FDR) [38] calculated based on the meta-p-values. Pathways with more than 500 associated entries were left out in this analysis for better interpretability. For both methods, the top-ranked pathways are the “alpha-Linolenic acid metabolism” (KEGG, 214 feature hits), the “jasmonic acid biosynthesis” (AraCyc, 176 feature hits), and the “lycolipid desaturation”(AraCyc, 325 feature hits). These pathways specifically describe parts of the biosynthesis of the well-known wound hormone jasmonate [39]. The first two pathways cover all biosynthetic steps from the fatty acid alpha-linolenic acid to jasmonic acid. The first committed step is catalyzed by the allene oxide synthase (AOS), whose gene is mutated in the *dde* 2-2 mutant plants [33]. The glycolipid desaturation pathway describes the formation of the alpha-linolenic acid via sequential steps of glycolipid-linked desaturation. The FDRs for these key pathways are much lower compared to the following pathways. Tables 4, 5, 6, 7, 8, 9, 10, and 11 show the results from enrichment analysis of the four single data sets and selected mappings of top-ranked features which were assigned to entries in the three key pathways, respectively. The enrichment analysis of the M1 data set (negative ionization mode, see Table 4) provides a major contribution to the results from meta-analysis. The first two pathways are also top-ranked but associated with much higher FDRs. The high-ranked features associated with jasmonic acid and its precursor metabolites, such as OPDA and OPC-8:0, are mainly responsible for this ranking (see Table 5). However, the mapping of putative monoisotopic feature masses to metabolites is error-prone and ambiguous. For example, OPDA, EOTrE, and a couple of other metabolites provided by KEGG and AraCyc share the same sum formula and single ion features cannot be unambiguously assigned without further information. In contrast to the alpha-linolenic acid metabolism pathway (KEGG), the very similar jasmonic acid biosynthesis pathway (AraCyc) is associated with a much higher FDR. This can be explained by a number of additional entries found only in the AraCyc version of the pathway and representing general substrates, such as acetyl-CoA, intermediate products which could not be measured with a high signal-to-noise ratio, such as OPC6-3-hydroxyacyl-CoA, or other side products. The glycolipid desaturation pathway, which can be found at position seven, is associated with a very high FDR. Most of the glycolipid species show higher intensities and signal-to-noise ratios in positive compared to negative ionization mode, which results in a very low FDR in pathway enrichment analysis of the M2 data set (see Tables 6 and 7). In contrast, jasmonate and many direct precursor metabolites cannot be measured in positive ionization mode with sufficient intensity, which explains the less prominent ranking of the alpha-linolenic acid metabolism (rank 12) and jasmonic acid biosynthesis (rank 13). Nonetheless, metabolites such as OPDA can be measured in both ionization modes with high signal-to-noise ratio and these findings confirm the corresponding pathways in meta-analysis. Integrating the Transcriptomics data sets T1 and T2 results in a much more comprehensive data interpretation (see Tables 9 and 11). Figure 4 exemplarily shows the pathway map of the alpha-linolenic acid metabolism with marked entries matched by high-ranking features from all data sets. In this combination, the ambiguous mapping of the MS data is supported by unambiguously matching transcripts. Almost all of the transcripts corresponding to enzymes in the alpha-linolenic acid metabolism can be found in the T1 and T2 data sets with relatively high signal-to-noise ratios. This results in much lower FDRs for the jasmonate-specific pathways in meta-analysis compared to the results from single Metabolomics data set analysis. Also in the analysis of the single Transcriptomics data sets (see Tables 8 and 10), these two pathways are associated with relatively high FDRs. In case of the T1 data set, both pathways can be found at less prominent positions (rank 14 and 23, see Table 8). For both Transcriptomics data sets, the glycolipid desaturation is ranked in the middle of all pathways (rank 420 and 161). Only a small number of transcripts associated with fatty acid desaturase show a high signal-to-noise ratio (see Tables 9 and 11).

**Table 2:** Results from meta-analysis of pathway enrichment (Brown's method).

Rank	DB	Pathway	Hits	KS	Rank-sum
1	KEGG	alpha-Linolenic acid metabolism	214	0.0001321	2.383e-05
2	AraCyc	jasmonic acid biosynthesis	176	0.003676	0.0003101
3	AraCyc	glycolipid desaturation	325	0.0007046	0.0102
4	KEGG	Linoleic acid metabolism	147	0.5252	0.3968
5	AraCyc	superpathway of phenylalanine, tyrosine and tryptophan biosynthesis	86	0.5364	0.5253
6	AraCyc	traumatin and (Z)-3-hexen-1-yl acetate biosynthesis	131	0.4538	0.5557
7	KEGG	2-Oxocarboxylic acid metabolism	578	0.5252	0.767
8	AraCyc	glucosinolate biosynthesis from dihomomethionine	153	0.5364	0.7857
9	KEGG	Starch and sucrose metabolism	335	0.5252	0.7857
10	KEGG	Proteasome	114	0.5252	0.7857

The table contains the high-ranking pathways from meta-analysis of pathway enrichment (Brown's method) based on the Kolmogorov-Smirnov (KS) and rank-sum test utilizing all data sets. The p-values per data set were restandardized. The pathways are sorted according to the meta-p-values derived from the rank-sum test. The second column (DB) contains the name of the source database, the fourth column (Hits) the number of feature assignments. The last two columns comprise the false discovery rates calculated from the meta-p-values.

**Table 3:** Results from meta-analysis of pathway enrichment (Stouffer's extended method).

Rank	DB	Pathway	Hits	KS	Rank-sum
1	KEGG	alpha-Linolenic acid metabolism	214	6.708e-05	1.127e-05
2	AraCyc	jasmonic acid biosynthesis	176	0.001774	7.328e-05
3	AraCyc	glycolipid desaturation	325	0.02043	0.2122
4	AraCyc	traumatin and (Z)-3-hexen-1-yl acetate biosynthesis	131	0.4545	0.4326
5	AraCyc	superpathway of phenylalanine, tyrosine and tryptophan biosynthesis	86	0.4545	0.4326
6	KEGG	Linoleic acid metabolism	147	0.7866	0.499
7	AraCyc	glucosinolate biosynthesis from dihomomethionine	153	0.4545	0.6282
8	AraCyc	glucosinolate biosynthesis from tryptophan	132	0.4545	0.6583
9	AraCyc	glucosinolate biosynthesis from phenylalanine	115	0.6522	0.6583
10	AraCyc	glucosinolate biosynthesis from tetrahomomethionine	114	0.4545	0.6583

The table contains the high-ranking pathways from meta-analysis of pathway enrichment (Stouffer's extended method) based on the Kolmogorov-Smirnov (KS) and rank-sum test utilizing all data sets. The last two columns comprise the false discovery rates calculated from the meta-p-values.

**Table 4:** Results from pathway enrichment analysis of data set M1.

Rank	DB	Pathway	Hits	KS	Rank-sum
1	KEGG	alpha-Linolenic acid metabolism	65	0.02084	0.03717
2	AraCyc	jasmonic acid biosynthesis	68	0.1531	0.1503
3	KEGG	Linoleic acid metabolism	43	0.4598	0.8524
4	AraCyc	indole-3-acetyl-amino acid biosynthesis	29	0.4598	0.8524
5	AraCyc	traumatin and (Z)-3-hexen-1-yl acetate biosynthesis	38	0.4598	0.8524
6	AraCyc	galactosylcyclitol biosynthesis	14	0.4598	0.8524
7	AraCyc	glycolipid desaturation	144	0.4598	0.8524
8	KEGG	Porphyrin and chlorophyll metabolism	222	0.8841	0.8524
9	AraCyc	poly-hydroxy fatty acids biosynthesis	59	0.9248	0.8524
10	KEGG	Lysine degradation	46	0.4598	0.8524

The table contains the high-ranking pathways from enrichment analysis of data set M1 based on the Kolmogorov-Smirnov (KS) and rank-sum test. The pathways are sorted according to the restandardized p-values derived from the rank-sum test. The last two columns comprise the false discovery rates calculated from the restandardized p-values.

**Table 5:** Selected feature mappings from data set M1.

Rank	rt	m/z	Mappings
1	0.73	255.1218	Jasmonic acid
3	0.73	209.1168	Jasmonic acid
7	0.73	256.1264	Jasmonic acid
8	2.08	337.1999	OPDA, EOTrE
11	2.08	338.2044	OPDA, EOTrE
321	5.66	986.6145	18:3/18:1-DGD, 18:2/18:2-DGD
324	5.78	822.5428	18:2/16:0-MGD, 18:1/16:1-MGD
410	5.53	820.5295	18:3/16:0-MGD, 18:2/16:1-MGD, 18:1/16:2-MGD
447	2.33	339.2155	OPC-8:0
540	5.64	960.5985	18:3/16:0-DGD
542	6.02	823.5541	18:1/16:0-MGD, 18:0/16:1-MGD
554	5.67	858.5064	18:3/18:3-MGD
563	5.74	795.5232	18:3/18:1-MGD, 18:2/18:2-MGD
650	6.18	939.5986	18:2/18:3-DGD
846	5.89	962.613	18:2/16:0-DGD
879	6.23	859.5155	18:2/18:3-MGD
899	1.86	309.2055	HpOTrE
1445	6.17	964.6258	18:1/16:0-DGD
1727	7.53	278.2245	Linolenic acid
2142	0.52	239.0895	9-Oxononanoic acid

The table shows selected mappings of features from data set M1 (24796 features) to entries in the first three pathways in tables 2 and 3. The first column contains the feature rank. The second and third column show the corresponding retention times and mass-to-charge ratios. Multiple mappings correspond to different ionization rules or isotopologues.

**Table 6:** Results from pathway enrichment analysis of data set M2.

Rank	DB	Pathway	Hits	KS	Rank-sum
1	AraCyc	glycolipid desaturation	167	0.0009173	0.002862
2	AraCyc	antheraxanthin and violaxanthin biosynthesis	63	0.1033	0.2477
3	KEGG	Carotenoid biosynthesis	389	0.3608	0.8365
4	AraCyc	zeaxanthin biosynthesis	29	0.5251	0.8365
5	AraCyc	lutein biosynthesis	34	0.5251	0.8365
6	AraCyc	capsanthin and capsorubin biosynthesis	38	0.5251	0.8365
7	AraCyc	brassinosteroids inactivation	20	0.5251	0.8365
8	KEGG	Porphyrin and chlorophyll metabolism	236	0.8693	0.8365
…	…	…	…	…	…
12	KEGG	alpha-Linolenic acid metabolism	89	0.8693	0.8365
13	AraCyc	jasmonic acid biosynthesis	54	0.8693	0.8365

The table contains the high-ranking pathways from enrichment analysis of data set M2 based on the Kolmogorov-Smirnov (KS) and rank-sum test. The last two columns comprise the false discovery rates calculated from the restandardized p-values.

**Table 7:** Selected feature mappings from data set M2.

Rank	rt	m/z	Mappings
2	2.08	310.2377	OPDA, EOTrE
8	2.08	293.2117	OPDA, EOTrE
11	2.08	311.2422	OPDA, EOTrE
48	2.08	315.1932	OPDA, EOTrE
180	6.17	942.6175	18:1/16:0-DGD
211	6.17	941.6124	18:2/18:3-DGD
231	6.22	772.5912	18:2/16:0-MGD, 18:1/16:1-MGD
248	5.51	776.5365	18:3/16:0-MGD, 18:2/16:1-MGD, 18:1/16:2-MGD
295	6.00	960.6576	18:3/18:1-DGD, 18:2/18:2-DGD
297	4.69	772.5034	18:3/16:2-MGD
310	5.07	937.5843	18:3/18:3-DGD
330	5.87	935.6452	18:2/16:0-DGD
413	6.15	915.5996	16:0/18:1-DGD
459	6.45	774.6054	18:1/16:0-MGD, 18:0/16:1-MGD
507	5.72	768.56	18:3/16:1-MGD, 18:2/16:2-MGD, 18:1/16:3-MGD
615	5.18	748.5052	18:3/16:3-MGD
699	1.41	441.3184	Volicitin

The table contains selected feature mappings from data set M2 (23325 features) to the first three pathways in tables 2 and 3. Multiple mappings correspond to different ionization rules or isotopologues.

**Table 8:** Results from pathway enrichment analysis of data set T1.

Rank	DB	Pathway	Hits	KS	Rank-sum
1	KEGG	Glycolysis/Gluconeogenesis	108	0.3527	0.2952
2	KEGG	Proteasome	57	0.2885	0.2952
3	KEGG	Protein processing in endoplasmic reticulum	176	0.2885	0.2952
4	KEGG	Ribosome	220	0.02489	0.2952
5	KEGG	Oxidative phosphorylation	118	0.2885	0.2952
6	KEGG	Phenylalanine, tyrosine and tryptophan biosynthesis	54	0.492	0.2952
7	AraCyc	superpathway of phenylalanine, tyrosine and tryptophan biosynthesis	43	0.4966	0.3302
…	…	…	…	…	…
14	AraCyc	jasmonic acid biosynthesis	27	0.8201	0.35
…	…	…	…	…	…
23	KEGG	alpha-Linolenic acid metabolism	30	0.6202	0.4782
…	…	…	…	…	…
420	AraCyc	glycolipid desaturation	7	0.976	0.9758

The table contains the high-ranking pathways from enrichment analysis of data set T1 based on the Kolmogorov-Smirnov (KS) and rank-sum test. The last two columns comprise the false discovery rates calculated from the restandardized p-values.

**Table 9:** Selected feature mappings from data set T1.

Rank	ID	Mappings
6	AT2G06050	12-oxophytodienoate reductase 3
12	AT3G11170	fatty acid desaturase 7
16	AT5G42650	allene oxide synthase
18	AT1G17420	lipoxygenase 3
82	AT2G06050	12-oxophytodienoate reductase 3
120	AT4G15440	hydroperoxide lyase 1
226	AT5G48880	peroxisomal 3-keto-acyl-CoA thiolase 5
241	AT2G44810	phospholipase A1
316	AT1G20510	OPC-8:0 CoA ligase 1
436	AT1G76680	12-oxophytodienoate reductase 1
638	AT1G72520	lipoxygenase 4
737	AT4G16760	peroxisomal acyl-coenzyme A oxidase 1
744	AT1G17420	lipoxygenase 3
1037	AT3G45140	lipoxygenase 2
1487	AT1G13280	allene oxide cyclase 4
2116	AT2G06925	phospholipase A2-ALPHA
2788	AT2G31360	delta 9 acyl-lipid desaturase 2
3146	AT3G15290	3-hydroxyacyl-CoA dehydrogenase
4263	AT5G04040	triacylglycerol lipase SDP1
4276	AT1G76150	enoyl-CoA hydratase 2

The table contains selected feature mappings from data set T1 (25392 features) to the first three pathways in tables 2 and 3. Multiple mappings correspond to different spots on the microarray.

**Table 10:** Results from pathway enrichment analysis of data set T2.

Rank	DB	Pathway	Hits	KS	Rank-sum
1	KEGG	alpha-Linolenic acid metabolism	30	0.5885	0.0794
2	KEGG	Starch and sucrose metabolism	142	0.6748	0.3277
3	AraCyc	jasmonic acid biosynthesis	27	0.7192	0.3277
4	KEGG	Linoleic acid metabolism	11	0.7192	0.7457
5	AraCyc	glucosinolate biosynthesis from phenylalanine	16	0.7192	0.7457
6	AraCyc	glucosinolate biosynthesis from dihomomethionine	19	0.7192	0.7457
7	KEGG	Valine, leucine and isoleucine biosynthesis	19	0.7192	0.7457
8	AraCyc	glucosinolate biosynthesis from tryptophan	21	0.7192	0.7457
9	AraCyc	starch degradation I	37	0.7192	0.7457
…	…	…	…	…	…
161	AraCyc	glycolipid desaturation	7	0.8851	0.9666

The table contains the high-ranking pathways from enrichment analysis of data set T2 based on the Kolmogorov-Smirnov (KS) and rank-sum test. The last two columns comprise the false discovery rates calculated from the restandardized p-values.

**Table 11:** Selected feature mappings from data set T2.

Rank	ID	Mappings
25	AT5G42650	allene oxide synthase
104	AT2G06050	12-oxophytodienoate reductase 3
355	AT1G76680	12-oxophytodienoate reductase 1
376	AT5G48880	peroxisomal 3-keto-acyl-CoA thiolase 5
426	AT1G17420	lipoxygenase 3
484	AT3G15870	oxidoreductase
631	AT1G19640	jasmonic acid carboxyl methyltransferase
1019	AT3G11170	fatty acid desaturase 7
1263	AT5G04040	triacylglycerol lipase SDP1
1354	AT4G16760	peroxisomal acyl-coenzyme A oxidase 1
1371	AT1G17420	lipoxygenase 3
1544	AT3G45140	lipoxygenase 2
1812	AT3G15850	fatty acid desaturase 5
1940	AT2G06925	phospholipase A2-ALPHA
2139	AT4G30950	fatty acid desaturase 6
2413	AT2G06050	12-oxophytodienoate reductase 3
2653	AT3G15290	3-hydroxyacyl-CoA dehydrogenase
3022	AT1G67560	lipoxygenase 3
3297	AT3G06860	3-hydroxyacyl-CoA dehydrogenase
3383	AT2G33150	peroxisomal 3-keto-acyl-CoA thiolase 2

The table contains selected feature mappings from data set T2 (25392 features) to the first three pathways in tables 2 and 3. Multiple mappings correspond to different spots on the microarray.

In case of both methods for meta-analysis, the pathways “Linoleic acid metabolism” and “traumatin and (Z)-3-hexen-1-yl acetate biosynthesis” can be found in the list of top-ten. These pathways are directly connected with the alpha-linolenic acid metabolism and affected by the AOS mutation as well [40]. However, it should be noted that the second pathway is only of limited relevance in this context because the used genotype Columbia is a natural mutant in its second enzymatic step, the fatty acid hydroperoxide lyase reaction [41]. The 2-Oxocarboxylic acid metabolism (Brown's method) and several pathways in the ranking based on Stouffer's extended method describe glucosinolate biosynthesis, the major chemical defense reaction of Arabidopsis plants upon wounding that is regulated by jasmonates [42]. Though, these pathways are associated with comparably high FDRs.

Comparing the results based on the KS and the rank-sum test, no clear trend towards lower FDRs can be observed. In case of Brown's method, the glycolipid desaturation pathway is associated with a much lower FDR for both tests. In case of Stouffer's extended method, both jasmonate-specific pathways are scored with lower FDRs.

## Discussion

The meta-analysis of pathway enrichment was evaluated and applied on two Metabolomics and two Transcriptomics data sets in the context of plant wounding. The meta-analysis based on Brown's and Stouffer's extended method is able to incorporate information from different independent and dependent omics data sets and distinguish key pathways in the experimental context. The FDRs calculated based on the meta-p-values are much lower compared to the single data set analysis. Especially for the pathway analysis of non-targeted Metabolomics studies, where the identification of metabolites is a bottleneck, the integration of data from other omics platforms, such as DNA microarrays, increases the value and reliability of results. In this application, Brown's and Stouffer's extended method showed overall similar results. However, Brown's method seems to be more powerful in case of pathways which are associated with extreme p-values for only a proportion of the data sets. The glycolipid desaturation pathway for example is associated with very small p-values (KS and rank-sum test) for the M2, relatively small p-values for the M1, and much larger p-values for the T1 and T2 data sets (see Table S1). In case of Brown's method, this pathway is associated with smaller FDRs (0.0007 and 0.01) in comparison to Stouffer's method (0.02 and 0.21). In contrast, Stouffer's method seems to be more powerful in case a pathway is associated with comparably small p-values for all data sets (see alpha-linolenic acid metabolism and jasmonic acid biosynthesis pathways). The choice of method depends on the objective of the meta-analysis, e.g. focus on pathways which show a consensus for all data sets or also including pathways with significant p-values for only a single or small number of data sets [26], [43]. In the context of heterogeneous omics data sets, which contain entities that cannot be measured in all experiments, e.g. metabolites that can be ionized either in positive or negative ionization mode, and pathways that may be associated with only a small number of entries for a particular omics platform, Brown's (or Fisher's method in case of independent p-values) seems to be the better choice. In both meta-analyses, a couple of pathways related to the wounding process were detected with relatively large FDRs. In order to combine the Metabolomics and Transcriptomics data sets in a robust way, we utilized general rank-based tests and a conservative restandardization of p-values per data set. The introduced framework may also be combined with more powerful tests specialized on microarray data analysis [37]. The enrichment analysis of the single T1 and T2 data sets resulted in considerably different rankings. This is likely to be related to the different time points when the wounded plants have been harvested (one and three hours).

In the performed simulation studies, the introduced feature rank correlation could be fully reconstructed utilizing the correlation estimation from pathway enrichment. By restricting the range of p-values via the parameter 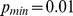, leaving out significant pathways, the estimation bias could be reduced. The comparison of the two dependent Metabolomics data sets, which were obtained from the same biological samples analyzed in positive and negative ionization mode, resulted in relatively small positive correlation coefficients. This indicates that only a small proportion of metabolites could be detected in both ionization modes with comparable quality of intensity profiles and that data from both modes should be considered in a comprehensive analysis. In general, the statistical power of the meta-analysis increases with decreasing dependence of data sets. Therefore, nearly independent data sets are desirable.

Comparing the one-sided KS and rank-sum test, both tests resulted in a similar distribution of normal deviates. In the simulation studies, the one-sided KS test was not able to fully reconstruct strong negative feature correlations. In most applications however, this type of data set correlation is not expected.

## Supporting Information

File S1 **Matlab source code for functions used in meta-analysis.**
(GZ) Click here for additional data file.

Dataset S1 **Data sets with database entry and pathway annotations.** The archive file contains the data sets in comma separated values format. The first column contains the feature IDs, respectively. The rt and Former m/z columns (M1 and M2 data set) contain the retention times and mass-to-charge ratios from MS analysis. The raw intensities for each sample can be found in the following columns. The s/n column shows the feature-specific signal-to-noise ratios and the last columns contain the KEGG and AraCyc entries and pathways mapped to the corresponding features and separated by slash characters.
(ZIP)

Table S1 **Pathways with p-values and FDRs from Pathway Enrichment Analysis.** The comma separated values file contains the p-values, restandardized p-values, meta-p-values, and corresponding FDRs for single data set and meta-analysis.
(CSV)

Table S2 **Supplementary tables for simulation studies.**
(PDF)

Technical Description S1 **Technical description of methods.**
(PDF)
