# Supplementary material for: Meta-Analysis of Pathway Enrichment: Combining Independent and Dependent Omics Data Sets
Source: PLoS One. 2014 Feb 28;9(2):e89297. doi: 10.1371/journal.pone.0089297 (PMC3938450; doi:10.1371/journal.pone.0089297)
Supplement: Table S2 — Supplementary tables for simulation studies. (PDF) [file pone.0089297.s004.pdf]

Table 1. Simulated positive correlation estimation from enrichment (KS test)

		p_{min}						
%	r_f	0	10^{-5}	10^{-4}	10^{-3}	0.01	0.05	0.1
0	1.00	1.00± 0.00	1.00± 0.00	1.00± 0.00	1.00± 0.00	1.00± 0.00	1.00± 0.00	1.00± 0.00
10	0.90	0.93± 0.02	0.93± 0.02	0.93± 0.02	0.92± 0.02	0.90± 0.02	0.86± 0.04	0.82± 0.05
20	0.80	0.87± 0.02	0.86± 0.03	0.85± 0.03	0.84± 0.03	0.80± 0.04	0.72± 0.05	0.66± 0.06
30	0.70	0.79± 0.03	0.78± 0.03	0.77± 0.04	0.75± 0.04	0.70± 0.05	0.60± 0.06	0.53± 0.07
40	0.60	0.71± 0.04	0.69± 0.05	0.68± 0.05	0.66± 0.05	0.60± 0.06	0.49± 0.07	0.41± 0.08
50	0.50	0.62± 0.05	0.60± 0.06	0.59± 0.06	0.56± 0.06	0.51± 0.06	0.40± 0.07	0.33± 0.08
60	0.40	0.52± 0.07	0.49± 0.07	0.49± 0.07	0.47± 0.08	0.40± 0.07	0.30± 0.08	0.24± 0.08
70	0.30	0.41± 0.08	0.38± 0.08	0.38± 0.08	0.36± 0.08	0.31± 0.08	0.22± 0.08	0.18± 0.09
80	0.20	0.28± 0.09	0.26± 0.09	0.25± 0.09	0.24± 0.09	0.21± 0.08	0.15± 0.08	0.11± 0.08
90	0.10	0.14± 0.10	0.13± 0.10	0.13± 0.10	0.12± 0.10	0.10± 0.08	0.07± 0.08	0.06± 0.09
100	0.00	-0.00± 0.10	-0.00± 0.10	-0.01± 0.10	0.00± 0.10	-0.00± 0.09	0.00± 0.08	0.01± 0.09

The table contains the average Pearson correlation coefficients calculated from enrichment analysis for the introduced positive correlation over all four data sets utilizing the Kolmogorov-Smirnov (KS) test for p-value calculation. The first column contains the percentage of randomly permuted feature ranks and the second column the average feature rank correlation coefficient as measurement of the introduced correlation. The other columns contain the average Pearson correlation coefficients calculated based on the transformed p-values from enrichment analysis for the given p_{min} and percentage value followed by the corresponding average sample standard deviation over 100 random permutations.

Table 2. Simulated positive correlation estimation from enrichment (rank-sum test)

		p_{min}						
%	r_f	0	10^{-5}	10^{-4}	10^{-3}	0.01	0.05	0.1
0	1.00	1.00± 0.00	1.00± 0.00	1.00± 0.00	1.00± 0.00	1.00± 0.00	1.00± 0.00	1.00± 0.00
10	0.90	0.96± 0.01	0.94± 0.01	0.94± 0.01	0.93± 0.02	0.91± 0.02	0.87± 0.04	0.83± 0.05
20	0.80	0.91± 0.02	0.88± 0.02	0.87± 0.03	0.86± 0.03	0.82± 0.04	0.75± 0.05	0.67± 0.07
30	0.70	0.85± 0.02	0.81± 0.03	0.80± 0.04	0.78± 0.04	0.72± 0.05	0.63± 0.07	0.54± 0.07
40	0.60	0.78± 0.04	0.73± 0.05	0.71± 0.05	0.69± 0.05	0.62± 0.06	0.52± 0.07	0.42± 0.09
50	0.50	0.69± 0.05	0.63± 0.06	0.62± 0.06	0.59± 0.06	0.53± 0.07	0.40± 0.08	0.32± 0.08
60	0.40	0.58± 0.07	0.53± 0.07	0.51± 0.07	0.48± 0.07	0.41± 0.08	0.32± 0.08	0.25± 0.09
70	0.30	0.48± 0.08	0.41± 0.09	0.39± 0.08	0.36± 0.08	0.31± 0.08	0.23± 0.07	0.17± 0.08
80	0.20	0.33± 0.09	0.28± 0.09	0.26± 0.09	0.25± 0.09	0.21± 0.08	0.14± 0.08	0.11± 0.09
90	0.10	0.17± 0.11	0.15± 0.11	0.14± 0.10	0.12± 0.10	0.11± 0.08	0.07± 0.08	0.06± 0.08
100	0.00	0.01± 0.11	0.00± 0.10	-0.00± 0.10	-0.01± 0.10	0.00± 0.08	-0.01± 0.09	0.00± 0.09

The table contains the average Pearson correlation coefficients calculated from enrichment analysis for the introduced positive correlation over all four data sets utilizing the rank-sum test for p-value calculation. The first column contains the percentage of randomly permuted feature ranks and the second column the average feature rank correlation coefficient as measurement of the introduced correlation. The other columns contain the average Pearson correlation coefficients calculated based on the transformed p-values from enrichment analysis for the given p_{min} and percentage value followed by the corresponding average sample standard deviation over 100 random permutations.

Table 3. Simulated negative correlation estimation from enrichment (KS test)

		p_{min}						
%	r_f	0	10^{-5}	10^{-4}	10^{-3}	0.01	0.05	0.1
0	-1.00	-0.78± 0.00	-0.80± 0.00	-0.79± 0.00	-0.78± 0.00	-0.75± 0.00	-0.66± 0.00	-0.58± 0.00
10	-0.90	-0.75± 0.02	-0.76± 0.02	-0.75± 0.02	-0.74± 0.02	-0.69± 0.03	-0.60± 0.04	-0.50± 0.05
20	-0.80	-0.71± 0.03	-0.72± 0.03	-0.70± 0.03	-0.69± 0.03	-0.64± 0.04	-0.52± 0.05	-0.43± 0.06
30	-0.70	-0.67± 0.04	-0.66± 0.04	-0.66± 0.04	-0.64± 0.04	-0.58± 0.05	-0.46± 0.06	-0.37± 0.07
40	-0.60	-0.61± 0.04	-0.61± 0.05	-0.60± 0.05	-0.58± 0.05	-0.52± 0.05	-0.40± 0.07	-0.30± 0.07
50	-0.50	-0.55± 0.06	-0.54± 0.06	-0.53± 0.06	-0.51± 0.06	-0.45± 0.06	-0.33± 0.07	-0.26± 0.08
60	-0.40	-0.47± 0.06	-0.46± 0.07	-0.45± 0.07	-0.43± 0.07	-0.37± 0.07	-0.26± 0.08	-0.19± 0.08
70	-0.30	-0.38± 0.07	-0.37± 0.08	-0.36± 0.08	-0.34± 0.08	-0.29± 0.07	-0.20± 0.08	-0.15± 0.08
80	-0.20	-0.26± 0.09	-0.26± 0.09	-0.24± 0.10	-0.24± 0.09	-0.20± 0.09	-0.12± 0.08	-0.10± 0.08
90	-0.10	-0.14± 0.10	-0.14± 0.09	-0.13± 0.10	-0.13± 0.10	-0.09± 0.09	-0.07± 0.08	-0.05± 0.08
100	-0.00	-0.00± 0.10	0.01± 0.10	-0.00± 0.10	-0.01± 0.10	0.00± 0.08	0.00± 0.08	-0.00± 0.08

The table contains the average Pearson correlation coefficients calculated from enrichment analysis for the introduced negative correlation over all four data sets utilizing the Kolmogorov-Smirnov (KS) test for p-value calculation. The first column contains the percentage of randomly permuted feature ranks and the second column the average feature rank correlation coefficient as measurement of the introduced correlation. The other columns contain the average Pearson correlation coefficients calculated based on the transformed p-values from enrichment analysis for the given p_{min} and percentage value followed by the corresponding average sample standard deviation over 100 random permutations.

Table 4. Simulated negative correlation estimation from enrichment (rank-sum test)

		p_{min}						
%	r_f	0	10^{-5}	10^{-4}	10^{-3}	0.01	0.05	0.1
0	-1.00	-1.00± 0.00	-1.00± 0.00	-1.00± 0.00	-1.00± 0.00	-1.00± 0.00	-1.00± 0.00	-1.00± 0.00
10	-0.90	-0.96± 0.01	-0.95± 0.01	-0.94± 0.01	-0.93± 0.02	-0.91± 0.03	-0.88± 0.04	-0.83± 0.05
20	-0.80	-0.91± 0.02	-0.88± 0.02	-0.87± 0.02	-0.86± 0.03	-0.82± 0.04	-0.75± 0.05	-0.67± 0.06
30	-0.70	-0.85± 0.03	-0.81± 0.03	-0.80± 0.04	-0.78± 0.04	-0.72± 0.05	-0.63± 0.07	-0.54± 0.08
40	-0.60	-0.78± 0.04	-0.73± 0.05	-0.71± 0.05	-0.69± 0.05	-0.63± 0.05	-0.52± 0.07	-0.41± 0.08
50	-0.50	-0.70± 0.05	-0.63± 0.06	-0.62± 0.06	-0.59± 0.06	-0.52± 0.07	-0.41± 0.08	-0.32± 0.08
60	-0.40	-0.59± 0.07	-0.53± 0.07	-0.51± 0.07	-0.47± 0.07	-0.42± 0.07	-0.31± 0.08	-0.25± 0.08
70	-0.30	-0.46± 0.08	-0.41± 0.09	-0.40± 0.09	-0.37± 0.08	-0.32± 0.08	-0.22± 0.09	-0.17± 0.08
80	-0.20	-0.33± 0.10	-0.29± 0.09	-0.27± 0.09	-0.26± 0.08	-0.21± 0.08	-0.15± 0.09	-0.11± 0.09
90	-0.10	-0.17± 0.11	-0.14± 0.10	-0.13± 0.09	-0.12± 0.09	-0.10± 0.09	-0.07± 0.08	-0.05± 0.09
100	0.00	0.01± 0.11	0.01± 0.10	0.01± 0.10	-0.00± 0.10	0.00± 0.09	0.00± 0.09	0.00± 0.08

The table contains the average Pearson correlation coefficients calculated from enrichment analysis for the introduced negative correlation over all four data sets utilizing the rank-sum test for p-value calculation. The first column contains the percentage of randomly permuted feature ranks and the second column the average feature rank correlation coefficient as measurement of the introduced correlation. The other columns contain the average Pearson correlation coefficients calculated based on the transformed p-values from enrichment analysis for the given p_{min} and percentage value followed by the corresponding average sample standard deviation over 100 random permutations.
