# Supplementary material for: Meta-Analysis of Pathway Enrichment: Combining Independent and Dependent Omics Data Sets
Source: PLoS One. 2014 Feb 28;9(2):e89297. doi: 10.1371/journal.pone.0089297 (PMC3938450; doi:10.1371/journal.pone.0089297)
Supplement: Technical Description S1 — Technical description of methods. (PDF) [file pone.0089297.s005.pdf]

Meta-Analysis of Pathway Enrichment: Technical Description

Data sets and preprocessing

For application and evaluation of the meta-analysis, two Metabolomics MS data sets [1] and two Transcriptomics DNA microarray data sets [2] were used. The Transcriptomics data sets can be obtained from the ArrayExpress [3] website (processed data, corrected cy3 and cy5 signals) under the IDs E-ATMX-9[1] and E-MEXP-1475[2]. The Metabolomics data sets can be downloaded from the MarVis homepage[3]. The feature profiles of all data sets were ranked separately utilizing a signal-to-noise ratio s/n (similar to the method described in [4]), where the signal s is calculated as the difference between the maximum and the minimum average condition-specific intensity over all conditions and the noise level n is defined as the pooled sample standard deviation (square root of the pooled sample variance) within all conditions.

Pathway Enrichment Analysis

The ranked features were mapped to the pathway entries in AraCyc[4] [5] and the Arabidopsis-specific pathways in the Kyoto Encyclopedia of Genes and Genomes (KEGG) database[5] [6]. In case of the Metabolomics MS data sets, all potential monoisotopic masses were calculated per feature based on the ionization rules and number of isotopes used in [1] and mapped to the metabolite masses in the databases using a tolerance of 0.005 Da. In case of the Transcriptomics DNA microarray data, the features were mapped to the *A. thaliana* genes utilizing their CATMA IDs [7]. Via this procedure, 17498 features from all data sets could be mapped to 663 different pathways. Based on the mappings, a set of feature ranks was extracted for each pathway and data set. In order to test for an over-representation of high-ranked features, a p-value was calculated for each set of ranks (pathway) utilizing a one-sided Kolmogorov-Smirnov (KS) or Wilcoxon rank-sum test (also known as Mann-Whitney U test) [8] as implemented in the MATLAB® kstest and ranksum functions (Statistics toolbox). In the first case, the empirical distribution of ranks in a given set is compared to the distribution of ranks in the respective data set. In the second case, the average rank of features in a given set is compared to the average rank for the whole data set without the features in the given set. The resulting p-values for the dependent Metabolomics data sets were used for the covariance estimation. The covariances between both Transcriptomics data sets and between the Metabolomics and Transcriptomics data sets, which were obtained from independent biological samples, were set to zero. Only pathways with less than 500 associated entries were subjected to enrichment and meta-analysis. For each pathway, only the p-values of data sets with at least one feature mapped to one of the pathway entries were considered.

Meta-analysis of independent p-values

In statistical meta-analysis, the most common methods for combining independent p-values from related tests are Fisher's [9] and Stouffer's method [10]. In Fisher's method, the meta-p-value for N independent p-values p_i is calculated based on the test statistic

$$T_F = -2\sum_{i=1}^{N} ln(p_i) \sim \chi^2(k = 2N) \tag{1}$$

which follows a chi-squared distribution with $2N$ degrees of freedom. In Stouffer's method, the test statistic is the sum of p-values transformed into normally distributed random variables (standard normal

[1] http://www.ebi.ac.uk/arrayexpress/experiments/E-ATMX-9/
[2] http://www.ebi.ac.uk/arrayexpress/experiments/E-MEXP-1475/
[3] http://marvis.gobics.de/data/wound_raw_data.zip
[4] Release biocyc-17.0, http://biocyc.org/
[5] KEGG FTP Release 2013-03-18, http://www.kegg.jp

deviates) based on the inverse cumulative distribution function:

$$T_S = \frac{\sum_{i=1}^{N} \Phi^{-1}(p_i)}{\sqrt{N}} \tag{2}$$

which is again standard normally distributed under the null hypothesis.

Meta-analysis of dependent p-values

For dependent p-values, a powerful approach is Brown's method [11], which is an extension of Fisher's method utilizing a known covariance matrix for standard normal deviates. The given p-values can be transformed into standard normal deviates by means of the inverse cumulative distribution function of the standard normal distribution. In order to incorporate the dependence of p-values, a scaled chi-squared distribution with a scaling parameter c and modified degrees of freedom f is assumed:

$$T_B \sim c\chi^2(k = f) \tag{3}$$

The parameters c and f are estimated based on the expected value and given covariance matrix of transformed p-values. For the calculation of the covariances (see formula 4 in [11]), the more precise estimation from [12] (formula 8) is used. The covariance matrix of the standard normal deviates $\Phi^{-1}(p_i)$ can also be utilized in order to extend Stouffer's method to dependent p-values assuming a multivariate normal distribution. In this case, the variance is calculated as sum of all covariances instead of using the number of p-values.

Estimation of covariances

In most applications with dependent data sets, the covariance matrix is not known and has to be estimated. In our proposed procedure, the pairwise covariance between two data sets is estimated based on the standard normal deviates of the pathway-specific p-values, which were obtained for each single data set in Pathway Enrichment Analysis. This estimation is expected to be biased by the alternative hypothesis since the similar or same experimental setup of the data sets imposes a certain dependence. In order to minimize this bias in the estimation of the pairwise covariance between two data sets i and j, a parameter p_{min} is introduced and only pathways with p-values $p_{min} < p_i, p_j < 1 - p_{min}$ are considered. Instead of directly estimating the sample covariance of the transformed p-values in this range (which would again be biased because of the range restriction), Pearson's correlation coefficient is used as normalized version of the sample covariance utilizing the sample mean and sample standard deviation for normalizing the data set-specific normal deviates. Finally, the estimated pairwise correlation coefficients are inserted into the covariance matrix of normal deviates. This is straight forward because the normal deviates were derived from the p-values based on the inverse cumulative distribution function of the standard normal distribution with unit standard deviation.

Simulated studies

The correlation estimation was evaluated by calculating the pairwise Pearson correlation coefficients between all four data sets and a copy of the respective data set with 0, 10, 20, ..., and 100 percent of the feature ranks randomly permuted. In order to generate the permuted copy of one of the data sets for a given percentage, a corresponding number of features were randomly selected and their ranks were randomly permuted. In contrast, the assignments of features to pathways were not modified. For each original and permuted data set, the p-values were calculated for all pathways using the KS or rank-sum test. The correlation coefficient between each original and permuted data set was computed based on the respective standard normal deviates (not restandardized) and the restriction of p-values utilizing different

parameter values $p_{min} = 0, 10^{-5}, 10^{-4}, 10^{-3}, 0.01, 0.05, 0.1$. For each percentage, p_{min} value, and data set, the random permutation and correlation estimation was repeated 100 times and the average correlation coefficient and sample standard deviation of correlation coefficients was calculated. As measurement of the introduced artificial correlation, the correlation coefficient between the feature ranks of each data set and the permuted ranks (feature rank correlation) was calculated and averaged, respectively. The whole procedure was repeated for negative correlation by randomly permuting a percentage of the inverted original feature ranks per data set. The results were averaged over all data sets.

References

1. Kaever A, Landesfeind M, Possienke M, Feussner K, Feussner I, et al. (2012) MarVis-Filter: Ranking, Filtering, Adduct and Isotope Correction of Mass Spectrometry Data. Journal of Biomedicine and Biotechnology 2012.

2. Yan Y, Stolz S, Chételat A, Reymond P, Pagni M, et al. (2007) A downstream mediator in the growth repression limb of the jasmonate pathway. The Plant Cell 19: 2470–2483.

3. Brazma A, Parkinson H, Sarkans U, Shojatalab M, Vilo J, et al. (2003) ArrayExpress-a public repository for microarray gene expression data at the EBI. Nucleic Acids Research 31: 68–71.

4. Tusher VG, Tibshirani R, Chu G (2001) Significance analysis of microarrays applied to the ionizing radiation response. Proceedings of the National Academy of Sciences 98: 5116–5121.

5. Mueller LA, Zhang P, Rhee SY (2003) AraCyc: a biochemical pathway database for Arabidopsis. Plant Physiology 132: 453–460.

6. Kanehisa M, Goto S, Sato Y, Furumichi M, Tanabe M (2012) KEGG for integration and interpretation of large-scale molecular data sets. Nucleic Acids Research 40: D109–D114.

7. Sclep G, Allemeersch J, Liechti R, De Meyer B, Beynon J, et al. (2007) CATMA, a comprehensive genome-scale resource for silencing and transcript profiling of Arabidopsis genes. BMC Bioinformatics 8: 400.

8. Barry WT, Nobel AB, Wright FA (2005) Significance analysis of functional categories in gene expression studies: a structured permutation approach. Bioinformatics 21: 1943–1949.

9. Fisher RA (1925) Statistical methods for research workers. Edinburgh: Oliver and Boyd.

10. Stouffer SA, Suchman EA, DeVinney LC, Star SA, Williams Jr RM (1949) The American soldier: adjustment during army life. Princeton: Princeton University Press.

11. Brown MB (1975) A method for combining non-independent, one-sided tests of significance. Biometrics 31: 987–992.

12. Kost JT, McDermott MP (2002) Combining dependent p-values. Statistics & Probability Letters 60: 183–190.
